# Selective serotonin re-uptake inhibitors affect craniofacial structures in a mouse model

**DOI:** 10.1371/journal.pone.0307134

**Published:** 2024-07-18

**Authors:** Quinn N. Saluan, George R. Bauer, Heema Vyas, Amr Mohi, Emily L. Durham, James J. Cray

**Affiliations:** 1 The Ohio State University College of Dentistry, Columbus, OH, United States of America; 2 Department of Biomedical Education and Anatomy, The Ohio State University College of Medicine, Columbus, OH, United States of America; 3 Division of Human Genetics, Department of Pediatrics, Children’s Hospital of Philadelphia, Philadelphia, PA, United States of America; Laboratoire de Biologie du Développement de Villefranche-sur-Mer, FRANCE

## Abstract

Selective serotonin re-uptake inhibitors (SSRI) widely used in the treatment of depression, anxiety, obsessive compulsive disorder, fibromyalgia, and migraine are among the most heavily prescribed drug class in the United States (US). Along with an overall rise in SSRI use, these medications are increasingly used by pregnant individuals and recent preclinical and clinical studies have indicated that SSRIs may increase the prevalence of congenital abnormalities and birth defects of the craniofacial region. Our group has developed pre-clinical models of study, including those that mimic the clinical use of SSRI in mice. Here we designed a study to interrogate a commonly prescribed SSRI drug, Citalopram, for its effects on craniofacial and dental development when introduced *in utero*. Pre-natal exposure to a clinically relevant dose of citalopram resulted in changes in craniofacial form identified by an increase in endocast volume in SSRI exposed postnatal day 15 mouse pups. More specifically, cranial length and synchondrosis length increased in SSRI exposed pups as compared to control pups of the same age. Additionally, growth center (synchondrosis) height and width and palate length and width decreased in SSRI exposed pups as compared to control un-exposed pups. Effects of SSRI on the molars was minimal. Craniofacial growth and development continue to be an area of interest in the investigation of *in utero* pharmaceutical drug exposure. Altogether these data indicate that prenatal SSRI exposure affects craniofacial form in multiple tissues and specifically at growth sites and centers of the skull.

## Introduction

Antidepressants, namely selective serotonin re-uptake inhibitors (SSRIs), are the most commonly used type of drug ages 20–59 [1]. In the last decade use of SSRIs has increased from 10.6% to 13.8% of the population in the United States (US) [2–4]. As adverse effects are relatively low with SSRIs, providers have widely used SSRIs as the drug of choice in developmentally vulnerable populations such as children, adolescents, and pregnant individuals [5–7]. In the past 12 years, antidepressant usage during pregnancy has increased 3% in Europe and 8% in the US [7]. Complex perinatal mood disorders, manifesting in various forms such as anxiety, bipolar affective disorder, and depression, have emerged amongst the most prevalent complications associated with pregnancy, [8]. The incidence of perinatal depressive disorder (PND) in the US is notably high, affecting approximately one in 7 to 10 pregnant women. Further, the mean rate of depression during the perinatal period is documented at 11.5% [9, 10]. If untreated, PND poses a significant risk to both maternal and infant health [11]. Existing literature underscores the adverse outcomes for neonates born to mothers with PND, including an increased likelihood of premature birth, lower fetal body weight, and reduced fetal body length [12–15]. To address these risks, clinicians frequently prescribe antidepressants, with SSRIs being a primary choice [2, 4].

Due to the surge of SSRI utilization among pregnant individuals, there has been an increase in research evaluating adverse effects these drugs may have on developing structures of the body. Recent evidence suggests that SSRIs may increase the prevalence of congenital abnormalities and birth defects particularly of the craniofacial complex [5, 6, 16]. Craniofacial development proceeds by combinatory influences of the expanding brain and oropharyngeal structure, osseous remodeling at the craniofacial growth sites (sutures), expansion of the cartilaginous growth centers of the skull (synchondroses) and often the mandibular joint cartilage [17–20]. Although much of the influence of growth proceeds under strict genetic and endocrine cues, alteration in development can occur due to genetic perturbation, environmental insult (teratogenesis) or altered gene-environment interactions disrupting homeostasis [21]. SSRIs readily cross the placenta and have been implicated in alterations to bone and cartilage development. Thus, these increasingly prescribed medications may pose a risk to the developing fetus [16, 22–25]. The once heavily prescribed SSRI, paroxetine (Paxil) was found to increase the risk of cardiac anomalies and has this been re-classified for pregnancy risk [26]. Studies concerning the effects of SSRIs in development are controversial and no consensus opinion is available. Although some SSRI drugs have noted contraindications when used by pregnant individuals [26, 27], the whole class of drugs does not have a designation as teratogens [28].

As craniofacial growth and development continues to be an area of interest in the investigation of *in utero* pharmaceutical drug exposure, our group has developed pre-clinical models of study, including those that mimic the clinical use of antidepressant drugs. Here we designed a study to interrogate Citalopram, a commonly prescribed SSRI drug that has shown similar properties to paroxetine [1], for its effects on craniofacial and dental development when introduced *in utero*. The *in utero* exposure window was chosen to capture the onset of depression during pregnancy where an SSRI regimen is started during pregnancy. Cephalometric and endocasting approaches determined segregating differences due to *in utero* SSRI exposure. Based on previous work investigating the impact of *in utero* SSRI exposure, our hypothesis is that craniofacial, palatal and dental morphology will be altered in individuals exposed *in utero* to SSRIs. These data will be useful in the continued interrogation of gene-environment interactions in craniofacial development to inform potential modulators of craniofacial homeostasis.

## Materials and methods

### Animal model and citalopram exposure

C57BL6 (Mus musculus, Jackson Laboratories, Bar Harbor, ME) breeding pairs (male and female mice 2–12 months old, n>3 respectively) were used to produce *in utero* exposed litters simulating fetal SSRI (citalopram, trade name Celexa) exposure. Citalopram was added to the drinking water of singly housed pregnant dams (~500 μg/day) from E13 to E20 in order to mimic a scaled clinically relevant dose of citalopram in humans. E13 was chosen as a starting point to allow for neurulation and neural crest cell formation to occur without drug insult to better study how structural outgrowth may be affected by these drugs when prescribed after pregnancy state is known. At birth, all treatment ceased. Dosage was identified as midrange for preclinical studies in murine models, was based upon an average consumption of 4 ml per day for a pregnant dam and was replaced as needed to provide *ad libitum* access [27]. Breeding animals were monitored daily by experienced research staff for signs of dehydration and distress. Control breeding pairs were provided drinking water without citalopram.

Resultant offspring were grown to postnatal day 15 (P15) at which point they were sacrificed using carbon dioxide asphyxiation with subsequent cervical dislocation and decapitation. Skulls were fixed in 4% paraformaldehyde for 48 hours and then switched to 70% ethanol for microcomputed tomography (μCT, described below) [2–4] Based on previous research, representative samples were used from our cohort of citalopram exposed (n = 25) and unexposed (n = 25). Care was taken to select individuals from multiple litters [2–4]. Animal Use Protocol was reviewed and approved by the Georgia Regents University Institutional Animal Care and Use Committee (2012–0498) and the Medical University of South Carolina Institutional Animal Care and Use Committee (AR#3510). All breeding procedures were carried out in an Association for Assessment and Accreditation of Laboratory Animal Care International accredited facility where all husbandry and related services are provided by the Division of Laboratory Animal Resources. All procedures and the reporting thereof comply with the Animal Research: Reporting *in Vivo* Experiments (ARRIVE) guidelines and included as S1 File [29].

### Morphometric analysis

**Fig 1:**
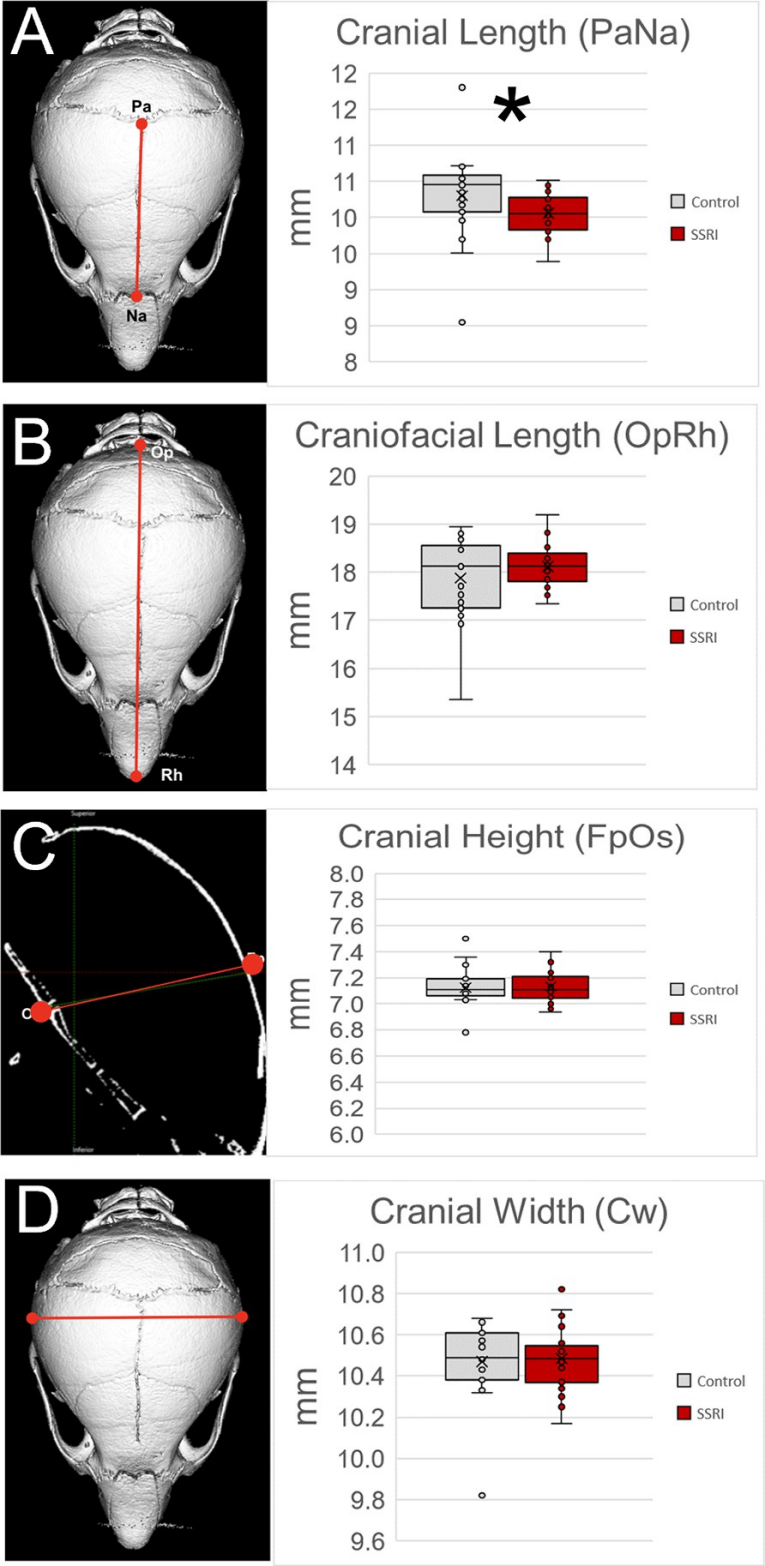
Cephalometric analysis of cranial form after *in utero* SSRI exposure. **A.** Depiction of cranial length measure and comparison between groups highlighting the decreased length in SSRI exposed individuals. Further analysis of craniofacial length (**B**), height (**C**), and width (**D**) indicated no other statistically significant differences between SSRI exposed and control groups. *p≤0.05 n = 25/group. Graphs represent standard box plots that are inclusive of identification of median (line inside box) a box representing first to third quartile range and whiskers in reference to the interquartile range and outliers that exist beyond the whiskers.

μCT images were obtained on P15 skulls using a SkyScan 1176 (Bruker Kartuizerseg 3B, 2550 Kontich, Belgium) scanner. Scans were collected on 50 animals using a previously published methodology and protocol [27, 30, 31]. For cephalometric analysis, 3D reconstructions of skulls were garnered with CTVox software v2.3.0 r810 (Skyscan) and measured using Analyze Pro (Analyze Direct, Overland Park, KY) and 3D slicer (slicer.org) [32] software. Skulls were numbered and investigators were blinded to exposure group during measurement and data acquisition. Endocast of rendered craniums were acquired using wrap solidify module of 3D Slicer after segmentation of bony tissue. Cephalometric analysis represented by 8 measurements was carried out via 3D rendered craniums and 2D image slices (**Table 1, Fig 1**).

**Table 1:** Measures and descriptions.

**Cephalometric Analysis** (3D Rendered Craniums)	
**PaNa**	Parietal point to nasion
**FpOs**	Fronto-parietal suture to occiput
**XPs**	Anterior sphenoid cranial base bone to posterior occipital cranial base bone
**Cw**	Cranial width; bilateral most lateral point of cranial vault
**OpRh**	Ophistion to rhinion
**Cranial Base Synchondroses** (2D μCT slice images)	
SOS_W_	Spheno-occipital synchondrosis width
SOS_L_	Spheno-occipital synchondrosis length (anterior-posterior)
SOS_H_	Spheno-occipital synchondrosis height
ISS_W_	Intersphenoidal synchondrosis width
ISS_L_	Intersphenoidal synchondrosis length (anterior-posterior)
ISS_H_	Intersphenoidal synchondrosis height
**Palate** (2D μCT slice images)	
Palate Length	Anterior sphenoid-ethmoid synchondrosis to the anterior-most aspect of the hard palate
Palate Width_A_	Anterior to M1 (midline left molar to midline right molar)
Palate Width_M_	Between M1 and M2 at midline (midline left molar to midline right molar)
Palate Width_P_	Posterior to M2 at midline (midline left molar to midline right molar)
**Molars** (2D μCT slice images)	
Molar Length	Anterior of M1 (midline) to posterior of M2 (midline)

**Fig 2:**
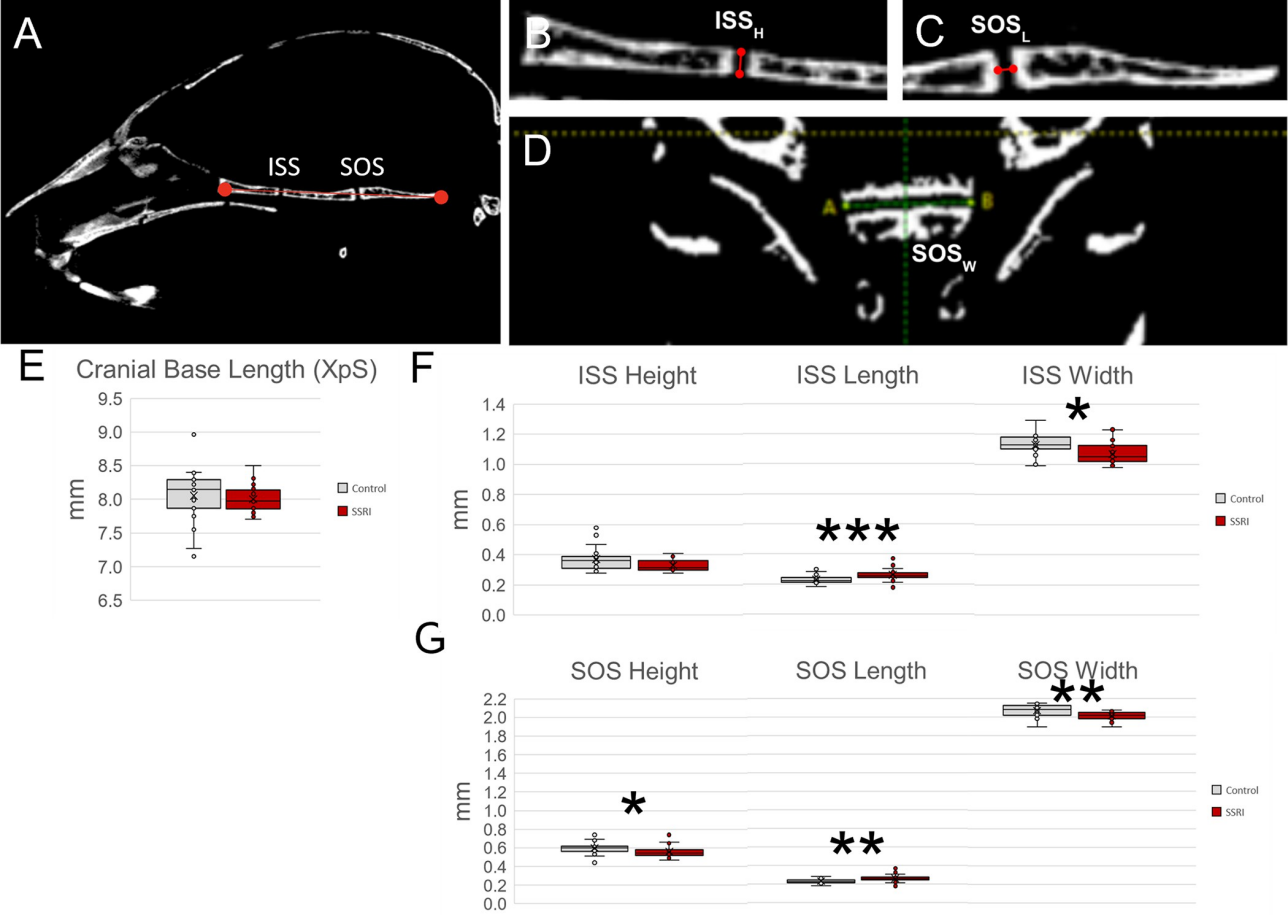
Analysis of cranial base form after *in utero* SSRI exposure. **A.** Depiction of cranial base length measure and comparison between groups (**E**) indicates no change in overall length due to SSRI exposure. Further analysis of the height, length, and width of ISS (**B-D, F**) indicates an increase in length, and a decrease in width of this synchondrosis in SSRI exposed pups. Likewise, investigation of the height, length, and width of the SOS indicates a decrease in both height and width and an increase in length due to in utero exposure to SSRI (**G**). Altogether, these data indicate that SSRI exposure affects the form of the growth centers of the skull. *p≤0.05, **p ≤0.01, ***p ≤0.001 n = 25/group. Graphs represent standard box plots that are inclusive of identification of median (line inside box) a box representing first to third quartile range and whiskers in reference to the interquartile range and outliers that exist beyond the whiskers.

Cranial base synchondroses, Spheno-Occipital Synchondrosis (SOS) and Inter-Sphenoidal Synchondrosis (ISS), were measured on 2D μCT images using Analyze Pro software. Synchondroses were measured in three dimensions: anterior-posterior length, width, and height. Length and height were measured on sagittal section and width was measured on coronal section (**Table 1, Fig 2**).

**Fig 3:**
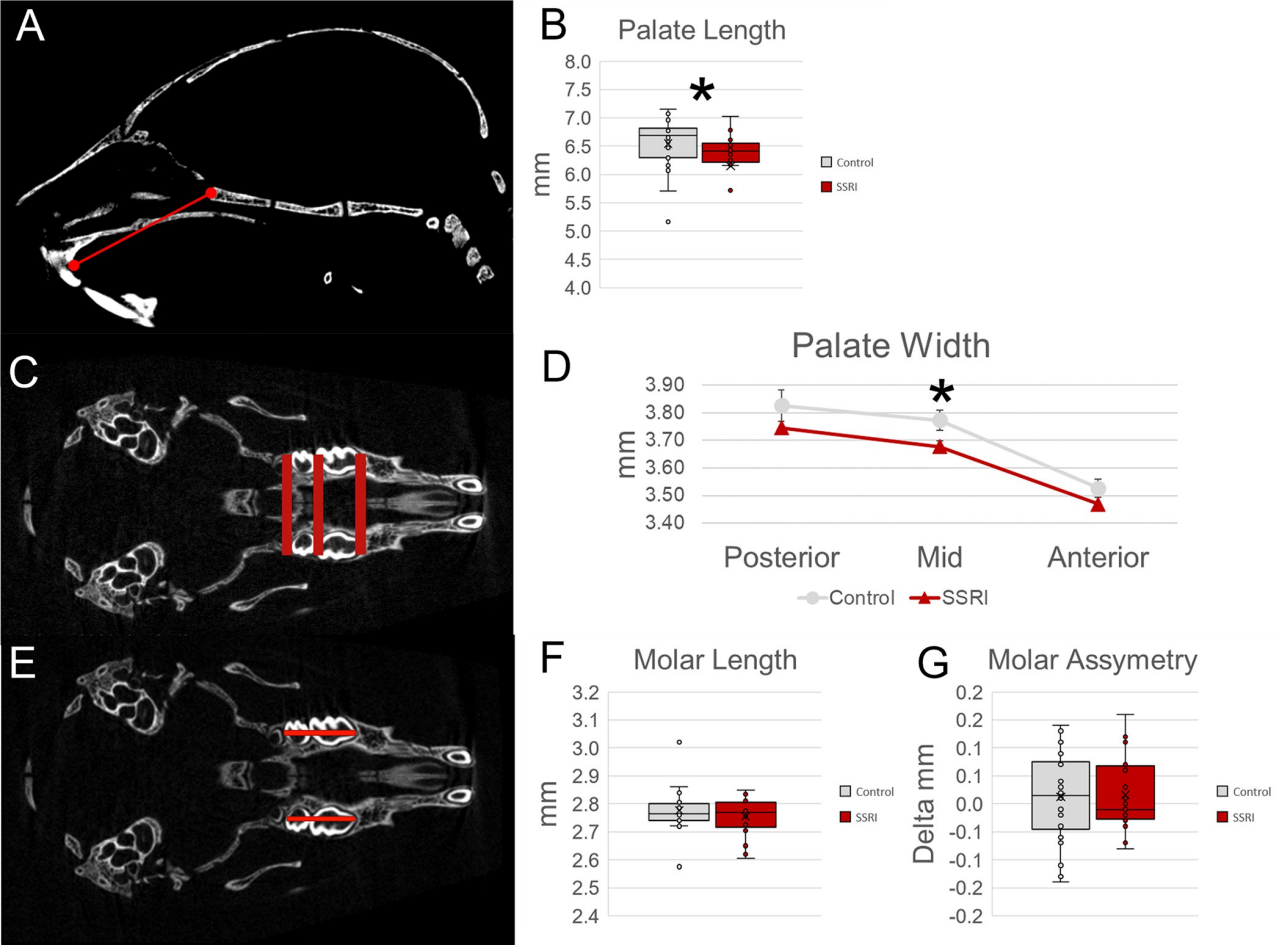
Oral cavity form after *in utero* SSRI exposure. **A.** Depiction of palate length measure and comparison between groups (**B**) indicates a decrease in palate length in SSRI exposed specimen potentially indicating that orthodontic concerns are appropriate. Further analysis of palatal width (**C**) also highlights an on average smaller (narrower) palate (**D**) in SSRI exposed specimen as compared to control. Interestingly, no changes in molar length or symmetry were identified due to in utero SSRI exposure (**E-G**). *p≤0.05, n = 25/group. Graphs represent standard box plots that are inclusive of identification of median (line inside box) a box representing first to third quartile range and whiskers in reference to the interquartile range and outliers that exist beyond the whiskers.

Within the oral cavity, palatal length was measured on sagittal 2D μCT images and width was measured on axial 2D μCT images. The palatal length was measured from the anterior sphenoid-ethmoid synchondrosis to the anterior-most aspect of the hard palate. Palatal width was measured in three parallel planes evenly distributed from anterior to posterior (**Table 1, Fig 3**). Murine maxillary molars were evaluated on axial section from anterior to posterior of the first and second molars (M1, M2) to measure overall length change in exposure versus control (**Table 1, Fig 3**).

### Statistical analysis

Our statistical approach focused on the differences between control and *in utero* SSRI exposed pups analyzed at postnatal day 15. All linear data was screened for normality (Shapiro Wilk Test) and homogeneity of variance (Browne Forsythe test). If assumptions were met, independent t-tests were utilized. Assumptions were met for the following analyses: Cranial Height, Cranial Base Length, Inter-Sphenoidal Synchondrosis Length, Spheno-Occipital Synchondrosis Width and Length, and Molar Assymetry. For all other analyses the assumptions were not met, and a non-parametric Mann Whitney U analyses was conducted. Raw data is provided in supplemental file (S1 Data). Differences were considered significant if p<0.05. All data was analyzed sing SPSS 28.0 (IBM, Inc.).

## Results

Cephalometric analyses revealed a statistically significant changes in skull dimensions. Cranial length (PaNa) was decreased in exposed pups as compared to control (*p* = 0.013**, Fig 1**). All other cephalometric analyses to assess width, height, and overall length of the skull were not significantly different between the two groups but of note was the overall increase in craniofacial length (OpRh, **Fig 1**). This measure was not found to be statistically significant but could at least partially account for the recorded increase in skull endocast volume.

Cranial base synchondroses were found to have changes in almost all dimensions due to *in utero* SSRI exposure. The SOS showed significant decreases in width and height in the SSRI exposed group (*p* = 0.003, *p* = 0.016) and an increase in length in the exposed group (*p* = 0.009, **Fig 2**). The ISS exhibited a significant increase in length in the exposed group (*p* < 0.001) and a significant decrease in width in the exposed group (*p* = 0.017, **Fig 2**).

Palatal dimensions were also found to have significant differences between the exposed and control groups. The palate length of the exposed group was significantly reduced (*p* = 0.016) compared to the control group. The overall width of the palate for the exposed group was found to be significantly reduced (*p* = 0.013) (**Fig 3**). The overall molar length and symmetry were found to have no significant difference between the exposed and unexposed groups (**Fig 3**).

**Fig 4:**
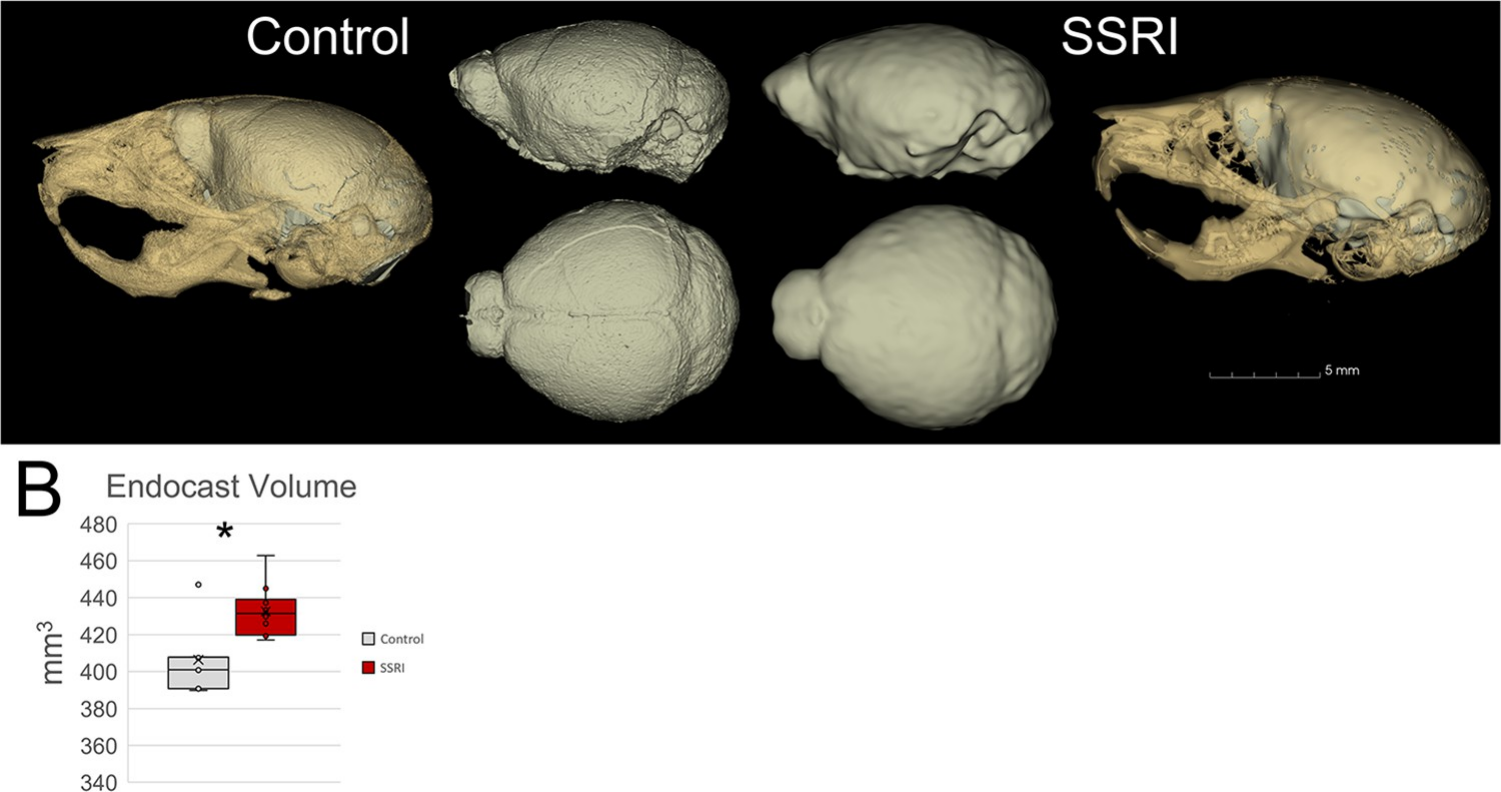
Change in skull form due to *in utero* SSRI exposure. **A.** 3D rendering of projected endocast (the space occupied by the brain) within skull (lateral view) with control on left and SSRI exposed on right. At center, direct comparison can be made between endocasts with lateral view top and superior view bottom and control on the left and SSRI on the right. **B**. Quantification and display of variability of endocast volume between control and SSRI (p = 0.0052) exposed P15 mouse pups. *p≤0.05 n = 10/group. Graphs represent standard box plots that are inclusive of identification of median (line inside box) a box representing first to third quartile range and whiskers in reference to the interquartile range and outliers that exist beyond the whiskers.

Finally, analysis of the volume occupied by the brain within the skull measured using an endocast projection (Wrap Solidify) in 3D slicer software indicates increased brain volume for SSRI exposed individuals (p = 0.0052, **Fig 4**).

## Discussion

Overall, our data corroborate that *in utero* exposure to Citalopram can cause significant deviations in craniofacial form [2, 3]. Our use of treated drinking water rather than other exposure models such as oral gavage replicates the varied SSRI dosage in the human population as well as avoided confounding stress variables. The space occupied by the brain in P15 mice exposed *in utero* to citalopram was larger than control accounting for the globular appearance of exposed skulls. This timepoint allowed for this assessment as the bone was developed enough to measure these changes in form. SSRIs have been found to specifically affect bone-related cells resulting in an overall decrease in bone in many cases [22, 27, 33–35]. This may also account for the increased endocast volume as bone may be thinner in SSRI exposed individuals (however we recorded no significant differences in bone volume between exposed and control skulls (p = 0.6049). Further, the decrease in skull length in conjunction with the noted increase in endocast volume could account for the globular appearance of the skull in mice exposed *in utero* to citalopram [2]. Dynamic changes in brain and skull shape track on one another regardless of changes in biomechanical and physiological function [18]. These data correlate with published data indicating changes in skull form after exposure to SSRI [27, 33, 36].

Changes in brain form may be caused by or may drive changes in craniofacial form. Thus, we conducted a cephalometric analysis post citalopram exposure. Our data suggest changes in the dimensions of cranial length, cranial base synchondroses SOS and ISS, and palatal length and width, partially supporting our hypotheses of alteration in growth due to *in utero* SSRI exposure. Importantly, two dimensional measures can obfuscate changes to 3 dimensional form. Assessments in the growth sites and centers of the skull indicate that exposure to SSRI may negatively affect growth and development of the head and face.

Previous studies have revealed that the dimension of the cranial base has an influence on different facial parameters such as maxillary prognathism [37]. This pattern is similar to that found in human with elongated cranial bases that present clinically with Class II facial structures [37, 38]. Here an overall decrease in cranial length (PaNa) coincides with dimension changes seen in the cranial base synchondroses. These cartilaginous growth centers increased in length and decreased in width as a result of *in utero* citalopram exposure. Growth centers at the base of the skull are noted to drive development of the face and head. Cranial base synchondroses showed consistency in their changes post-exposure, namely an overall increase in length and decrease in width. These findings point to longer yet narrower synchondroses with the SOS showing a decrease in height as well. Of note but not statistically significant was the overall increase in craniofacial length (OpRh), which suggests some compensatory growth in the anterior to posterior dimension of the overall skull [37]. As the synchondroses were particularly affected as evidenced by our microanatomical measures, it is worth noting future studies by our group will likely focus on the specific effects SSRIs might have on cartilaginous cell cycle and extracellular matrix production (collagens and GAGS) to determine if SSRIs target cartilage cells.

The cephalometric analysis of the palate indicated significant changes to the shape evidenced by a shorter narrower palate. The shorter and narrower palate may be an indication of changes in growth trajectory due to *in utero* SSRI exposure that is not compensated for by growth at the midpalatal sutures or in the periphery. This data is also confirmatory as previous studies have found a similar reduction in skull size and snout length in mice exposed to similar doses of citalopram [24]. Recent studies have also found that mice exposed to sertraline *in utero* have an increased risk of developing cleft palate [39]. Although no clefting was observed here, narrow and short palates are frequently encountered in orthodontic treatment and do represent variation that requires clinical intervention. Murine dentition was not affected by the exposure and remained otherwise consistent. Overall, these data suggest specific anatomical areas may be more affected by *in utero* SSRI exposure.

As much research in connective tissues and SSRIs have focused on co-morbidities in osteoporosis and target cells of osteoblasts and osteoclasts, future assessments should include cartilaginous regions and collagen organization. These assessments will be important in the context of Post Traumatic Stress Disorder where SSRIs are a first line intervention for veterans [40]. In addition, when cognitive-behavioral therapy is unavailable, this assessment becomes crucial for adolescents prescribed SSRIs to address conditions such as depression, obsessive-compulsive disorder, and post-traumatic stress disorder [41]. Further, this more tissue specific assessment will provide a novel direction to better understand perturbations in development. The direct effects of serotonin or SSRI exposure during development should be explored including the role of the serotonin (5-HT) receptor in cell migration, gene expression, and cell differentiation of the craniofacial region, specifically the oral cavity and at the base of the skull.

## Conclusion

Overall, these results indicate changes in bony structures such as the palate and cranium but also most notably changes in cartilaginous structures such as the cranial base synchondroses due to *in utero* SSRI exposure. Of interest is that SSRIs have been shown to increase cartilage degeneration which if these processes occur early enough in development, could disrupt growth [23, 36]. Together these data, the reclassification of some SSRIs to Class C (not appropriate for pregnant people), and the increasing frequency by which these medications are prescribed indicate an immediate need for more investigation into the mechanisms by which SSRIs disrupt craniofacial structural development.

## Supporting information

S1 File (PDF)

S1 Data (XLSX)
